# Transcriptome Analysis of Protection by *Dendrobium nobile* Alkaloids (DNLA) against Chronic Alcoholic Liver Injury in Mice

**DOI:** 10.3390/biomedicines10112800

**Published:** 2022-11-03

**Authors:** Xianyu Huang, Shan Yang, Jian Sun, Xia Li, Shao-Yu Zhou, Jing-Shan Shi, Jie Liu, Qin Wu

**Affiliations:** Key Laboratory of Basic Pharmacology of Ministry of Education and Joint International Research Laboratory of Ethnomedicine of Ministry of Education, Zunyi Medical University, Zunyi 563006, China

**Keywords:** chronic alcohol liver injury, *Dendrobium nobile* Lindl. alkaloids (DNLA), alcohol-related fatty liver, RNA-Seq, ingenuity pathway analysis, gene expression

## Abstract

Objective: To investigate the protective effects of *Dendrobium nobile* Lindl. alkaloids (DNLA) against chronic alcoholic liver injury. C57BL/6J mice were fed with the Lieber–DeCarli alcohol diet to induce chronic alcoholic liver injury. DNLA (20 mg/kg/day) was gavaged along with the alcohol diet for 28 days. Liver injury was evaluated by serum enzymes. Triglyceride levels, histopathology, and transcriptome changes were examined by RNA-Seq and qPCR. DNLA decreased serum triglyceride levels in mice receiving alcohol. Hepatocyte degeneration and steatosis were ameliorated by DNLA, as evidenced by H&E and Oil-red O staining. DNLA brought the alcohol-induced aberrant gene expression pattern towards normal. Alcohol induced 787 differentially expressed genes (padj < 0.01). DNLA induced 280 differentially expressed genes to a much less extent. Ingenuity pathway analysis showed that DNLA ameliorated alcohol-induced oxidative stress and xenobiotic metabolism disruption. qPCR verified that DNLA alleviated over-activation of Cyp2a4, Cyp2b10, and Abcc4; attenuated oxidative stress (Hmox1, Gstm3, Nupr1), reduced the expression of Nrf2 genes (Nqo1, Gclc, Vldlr); and rescued some metabolic genes (Insig1, Xbp1, Socs3, Slc10a2). In conclusion, DNLA was effective against alcohol-induced fatty liver disease, and the protection may be attributed to alleviated oxidative stress and restored metabolism homeostasis, probably through modulating nuclear receptor CAR-, PXR-, and Nrf2-mediated gene expression pathways.

## 1. Introduction

Binge drinking is a global problem, and alcohol-associated liver diseases (ALD) comprise a substantial portion of liver diseases, and they have high mortality rates [1,2]. Alcohol is mainly metabolized in the liver. In the cytoplasm, alcohol is metabolized to acetaldehyde, mainly via alcohol dehydrogenase (ADH), and then processed by aldehyde dehydrogenase (ALDH) in mitochondria to produce acetate/acetyl CoA [3]. The microsomal ethanol oxidizing system is closely involved in the ethanol disposition. Cytochrome P450 2E1 (Cyp2e1), and enzyme located in the endoplasmic reticulum, plays a critical role in the generation of reactive oxygen species from alcohol metabolism, leading to oxidative stress in the liver and subsequent induction of Cyp2a5 and the Nrf2 pathways as adaptive responses [4]. Chronic alcoholic liver injury is characterized by steatosis, which can further develop to alcoholic hepatitis, fibrosis, cirrhosis, and even hepatocellular carcinoma with continuous consumption of excessive alcohol [1].

Combination therapy options including the use of traditional medicine [5,6] towards multiple targets will eventually be required for ALD [7]. Dendrobium species have been widely used as Chinese medicines; they have many beneficial effects [8]. *Dendrobium nobile* Lindl. is the most popular herbal medicine in Guizhou, China, and is included in the Chinese Pharmacopoeia [9]. At least 82 active ingredients have been isolated from *Dendrobium nobile* Lindl., including alkaloids, glycosides, polysaccharides, phenanthrene, dibenzyl compounds, and others [10]. *Dendrobium nobile* Lindl. alkaloids (DNLA) are the main bioactive components of Dendrobium and have been proven to have a variety of beneficial activities, including neuroprotective effects against Alzheimer’s disease models [11] and Parkinson’s disease models [12]. They have anti-aging, anti-diabetes, and anti-hyperlipidemia properties [10]. DNLA can regulate hepatic metabolism gene expression [13] and affect the lipid homeostasis of the liver via regulating bile acid metabolism [14]. Furthermore, DNLA has been demonstrated to exhibit protective effects on liver injury induced by CCl_4_ in wild-type mice, but not in Nrf2-null mice, indicating the activation of the Nrf2/ARE pathway as a mechanism of protection [15]. DNLA improves CCl_4_ -induced mitochondrial dysfunction and mitochondrial oxidative stress in an Nrf2-dependent manner [16]. RNA-Seq analysis of DNLA protection against CCl_4_ hepatotoxicity revealed multiple molecular targets contributing to the protection [17]. However, little is known about the effects of DNLA on alcoholic liver diseases. 

The risks associated with chronic alcoholic liver injury should not be underestimated. Other than stopping drinking, there are limited therapeutic strategies to reverse alcoholic liver injury. Therefore, discovering therapeutic candidates for the treatment of alcoholic liver injury from Chinese herbal medicine would be significant [5,6,18,19,20] and of clinical significance. In this study, a chronic liver injury model was induced by the Lieber–DeCarli liquid diet containing 5% alcohol [21], and the effects of DNLA intervention were evaluated after 28 days of treatment. RNA-Seq and qPCR were utilized to explore the molecular mechanism of DNLA-induced protection. The results revealed promising efficacy and novel molecular targets of DNLA to ameliorate chronic alcoholic liver injury.

## 2. Materials and Methods

### 2.1. Materials

The original drug of *Dendrobium nobile* was purchased from Xintian Pharmaceutical Ltd. (Chishui, Guizhou, China). Total alkaloids were extracted by Chengchen Zhang of the Key Laboratory of Basic Pharmacology of the Ministry of Education of Zunyi Medical University. The proportion of DNLA was 78.9% according to LC-MS, including dendrobine, dendrobine-N-oxide, nobilonine, dendroxine, 6-hydroxynobilonine and 13-hydroxy-14-oxodendrobine, as previously described [22,23]. DNLA was prepared in 1% tween-80 and stored at 4 °C. 

### 2.2. Animals and Treatment

Adult, 8-week-old, male C57BL/6 J mice were purchased from Zhejiang Vital River Experimental Animal Technology Co., Ltd. (Jiaxing, China) (license number: SCXK2021-0006) and maintained in a specific pathogen-free (SPF) animal room (temperature 22 ± 2 °C, humidity 50 ± 10%, 12 h light/dark cycle), with free access to food and water, at the Key Lab of Basic Pharmacology, Zunyi Medical University. Animal handling was in full compliance with the Chinese Experimental Animal Guideline and approved by the Animal Ethics Committee of Zunyi Medical University (ZMU21-2105-002) on 16 May 2021. 

After five days of acclimation, all mice were randomly divided into the following three groups: pair-fed, EtOH-fed, and EtOH-fed+DNLA (n = 9). Pair-fed mice received the liquid control diet without alcohol. After adaptation to a gradual increase in alcohol from 1% to 5% in the Lieber–DeCarli liquid diet for 5 days, both EtOH-fed and EtOH-fed+DNLA mice were kept on the Lieber–DeCarli liquid diet containing 5% alcohol for 28 days. From the sixth day, pair-fed and EtOH-fed groups were gavaged with 10 mL/kg/d of normal saline, and EtOH-fed+DNLA mice were gavaged with DNLA (20 mg/kg). The dose of DNLA was selected based on our previous dose–response studies [15,16,17]. Four hours after the last dosing, mice were euthanized; blood and livers were collected.

### 2.3. Histopathology Examination

A portion of the liver was fixed with 10% paraformaldehyde for 48 h, then embedded in paraffin wax. The samples were cut into 3.5 μm slices using a Leica RM2235 microtome (Leica, Wetzlar, Germany) and subsequently processed in standard histology procedures for H&E staining. Other portion of the liver was embedded in optimal cutting temperature compound (OCT) and stored at −80 °C. The samples were sectioned into 4 μm slices using a freezing microtome (Leica, Germany) for subsequent Oil-red O staining to evaluate lipid droplets. Three photographs were randomly taken from each sample using an Olympus microscope (Olympus Corporation, Tokyo, Japan).

### 2.4. Serum Enzyme Activities and Triglyceride Levels

Blood was centrifuged at 3000 rpm for 10 min to obtain the serum. Serum alanine transaminase (ALT), aspartate transaminase (AST) and triglyceride (TG) were determined following the instructions of corresponding biochemical kits (Nanjing Jiancheng, Nanjing, China). The absorbance was measured by the Multiskan Go Full Wavelength Microplate Reader (Thermo Fisher, Waltham, MA, USA).

### 2.5. RNA Isolation and Sequencing 

Total RNA of liver tissue was extracted by Trizol reagent (Takara, Japan). The quantity and quality of RNA were determined by the NanoDrop 2000 Ultra micro-spectrometer (Thermo Fisher, USA). RNA samples were reverse-transcribed with Oligo dT primer to produce cDNA. The generated first-strand cDNA was co-reacted with the RNase H enzyme, DNA polymerase and T4 ligase to generate double-stranded cDNA; the double-stranded cDNA was fragmented by the Tn5 enzyme, and we added the remedial design (RD) sequence required for sequencing at both ends. Sequencing primers at both ends of P5 and P7 were connected by RD sequences at both ends, and enrichment PCR amplification was performed. Successful library construction was sequenced. Finally, bioinformatic analyses of differentially expressed genes from RNA-Seq data between groups were performed by Chongqing Weilang Biotechnology Co. Ltd. (Chongqing, China). 

### 2.6. Bioinformatic Analysis

Principal component analysis (PCA) was performed to visualize the pattern of distribution by Partek Flow (Partek Inc., St. Louis, MO, USA). Total original gene counts of RNA sequencing of 9 samples (about 22,500 per sample) were imported into the Partek Flow Server. The distribution differences among groups were visualized.

Ingenuity pathway analysis (IPA) is a web-based bioinformatics application that allows researchers to upload RNA-Seq data for core analysis and comparative analysis to further understand biological effects (Qiagen, Redwood City, CA, USA). Canonical pathway and upstream regulator analyses were performed. The Z-scores were used to evaluate changes between EtOH-fed and pair-fed and EtOH-fed+DNLA and pair-fed groups. 

Differentially expressed genes analysis (DEGs) was performed with the DESeq2 method and the pair-fed group as the control. Padj < 0.01 was considered as statistically significant. Two-dimensional, hierarchical complete linkages of DEGs were generated by The Gene Cluster version 3.0 (https://cluster2.software.informer.com/3.0/ (accessed on 15 January 2022). Then, the CDT files were processed by TreeView version 1.6 (https://treeview.software.informer.com/1.6/ (accessed on 15 January 2022)) to generate the heatmap.

### 2.7. Quantitative Real-Time PCR

Total RNA was quantified at 260/280 nm and adjusted to 100 ng/μL. Reverse transcription was performed by Prime script RT Reagent Kit instructions (Takara Bio, Tokyo, Japan). The PCR reaction system was 15 μL (SYBR Green Supermix, 7.5 μL; 5 uM primer mix, 2 μL; and DEPC water, 2.5 μL). The primers were designed by Primer3 and synthesized by Sangon Biotech (Shanghai, China) (Supplementary Table S1). The reaction was performed with a CFX 96 real-time Fluorescence Quantitative PCR instrument (Bio-Rad, Hercules, CA, USA) using the iTaq Universal SYBR Green Supermix (Takara Bio, Japan). The relative mRNA levels were normalized with GAPDH of each sample and calculated using the 2^−△△CT^ formula. 

### 2.8. Western Blot

Total protein was extracted from the liver samples with RIPA lysis buffer containing 1 mM PMSF and proteinase inhibitors (Beyotime, Shanghai, China). The protein concentration was quantified by a BCA protein assay kit (buffer (Biotime, Shanghai, China). An aliquot of 30 μg of protein was denatured at 95 °C for 10 min and electrophoretic ally separated on 10% gradient sodium dodecyl sulfate polyacrylamide gel electrophoresis (SDS-PAGE) and transferred to a polyvinylidene difluoride (PVDF, 0.45 μm) membrane (Millipore, Billercia, MA, USA). The membranes were blocked with 5% defatted milk in TBST buffer (150 mmol/L NaCl, 50 mmol/L Tris−HCl (pH∼7.6), 0.1 % Tween-20) for two hours at room temperature, and incubated with anti-CYP2B10 polyclonal antibody (sc73456, 1:1000, Santacruz Biotechnology, Santa Cruz, CA, USA), anti-PXR polyclonal antibody (bs2334R, 1:1000, Bioss, Woburn, MA, USA), anti-HO-1 monoclonal antibody (ab85309, 1:1000, Abcam, Waltham, MA, USA), anti-NQO1 monoclonal antibody (ab28947, Abcam, Cambridge, MA, USA), anti-GCLC monoclonal antibody (ab190685, 1:1000, Abcam, USA), or anti-β-actin monoclonal antibody (1:5000, ProteinTech Group, Wuhan, China) at 4 °C overnight. Next, membranes were washed with TBST three times, and the membranes were probed with appropriate horseradish peroxidase-conjugated secondary antibodies (1:5000) for 1 h at room temperature. The membranes were visualized using chemiluminescence reagent BeyoECL Plus (Beyotime, Shanghai, China). The image was scanned, and band densities were quantified using Quantity One 1D analysis software v4.52 (Bio-Rad, Hercules, CA, USA). β-actin was used to normalize protein loading. 

### 2.9. Statistical Analysis

Differentially expressed genes (DEGs) from RNA-Seq were analyzed by DeSeq2 setting padj < 0.01 compared to pair-fed (*p*-value after Benjamini and Hochberg correction). The data of ALT, AST, triglyceride, and qPCR are expressed as mean ± SEM. One-way ANOVA was used to determine the statistical differences among groups, followed by Dunn’s multiple range tests. The significance level was set to *p* < 0.05 for all comparisons. 

## 3. Results

### 3.1. DNLA Protected against Chronic Alcohol-Induced Fatty Liver Disease

The effects of DNLA on bodyweight and liver index

**Figure 1:**
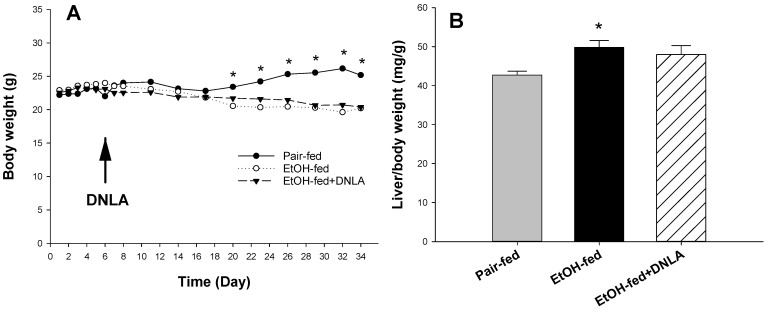
The effects of DNLA on mouse bodyweight and liver index. (**A**), Comparison of mouse bodyweights of different groups; (**B**) The effects of DNLA on liver/bodyweight. Data are mean ± SEM (n = 9). * Significantly different from the pair-fed group, *p* < 0.05.

The changes in bodyweight in each group over time are shown in Figure 1A. Differences in bodyweight between the EtOH-fed and the pair-fed groups were significant. Compared with the pair-fed group, the weight of mice in the EtOH-fed group decreased from the 20th day of alcohol consumption. DNLA treatment did not alter the decline of bodyweight. Alcohol feeding increased liver/bodyweight ratio, and DNLA had no effect on the liver/bodyweight ratio (Figure 1B).

The effects of DNLA on serum enzyme activity and triglyceride levels

**Figure 2:**
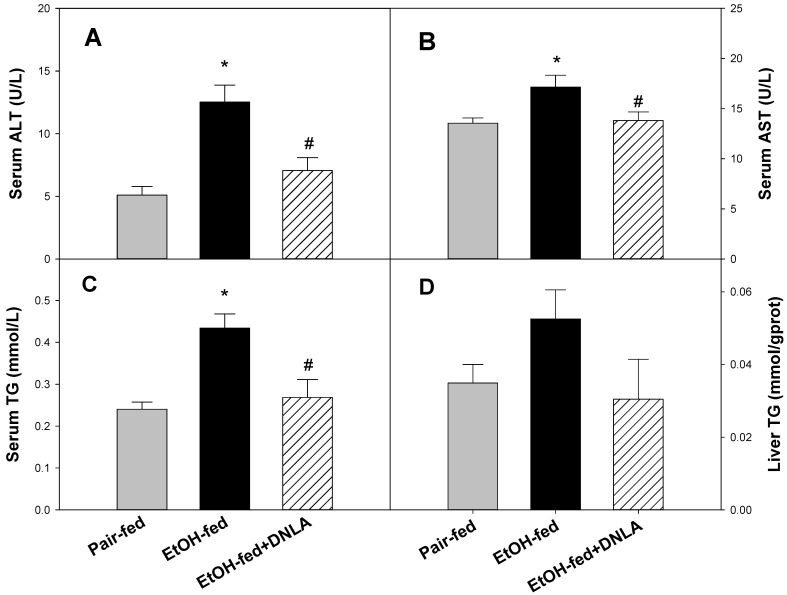
Serum activities of alanine aminotransferase (ALT, (**A**)), aspartate aminotransferase (AST, (**B**)), serum triglyceride (TG) (**C**), and liver TG (**D**) levels. Data are mean ± SEM (n = 9). * Significantly different from pair-fed, *p* < 0.05. # Significantly different from EtOH-fed, *p* < 0.05.

Serum enzyme activity results showed that continuous alcohol feeding increased alanine aminotransferase (ALT) (Figure 2A) and aspartate aminotransferase (AST) (Figure 2B) levels in serum, whereas DNLA decreased the ALT and AST levels in serum. 

The levels of serum triglyceride (TG) levels in the EtOH-fed group increased by 81%, and DNLA lessened this increase (Figure 2C). Liver triglyceride levels increased 50%, and DNLA alleviated this increase. However, the effects were not statistically significant due to greater individual variations (Figure 2D). 

### 3.2. DNLA Improved Alcohol-Induced Histopathology

**Figure 3:**
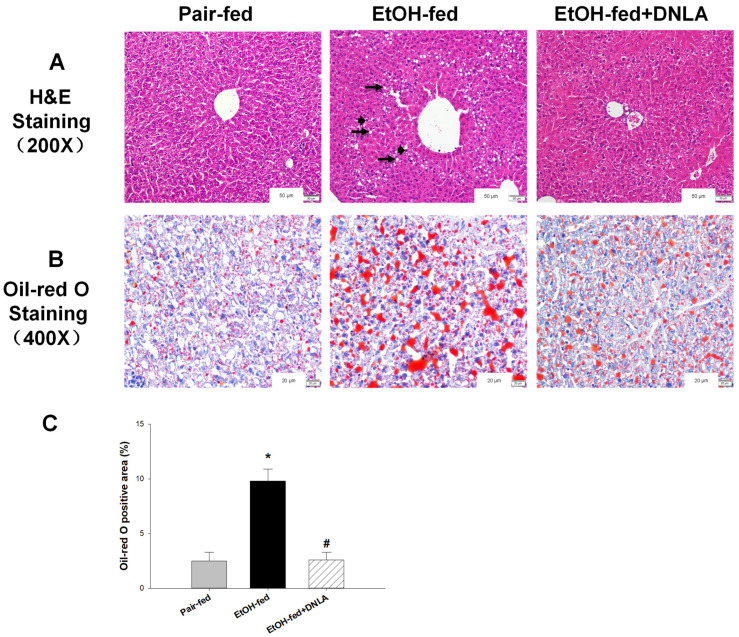
Histopathological examination of liver tissues. (**A**) Representative photograph of hematoxylin–eosin staining (H&E) (200×, with enlarged scale label) of liver tissues of different groups. Arrows indicate balloon-like degeneration and vacuole-like denaturation; arrowheads indicate foci of apoptotic/necrotic cells. (**B**) Representative images showing Oil-red O staining (400×). Red indicates lipid droplets accumulation. (**C**) The quantitation of percentage of Oil-red O-stained area. * Significantly different from pair-fed, *p* < 0.05. # Significantly different from EtOH-fed, *p* < 0.05.

In the pair-fed group, the morphology and structure of the liver were intact, and hepatocytes were neatly arranged, as evidenced by H&E-staining (Figure 3A). Compared to pair-fed mice, balloon-like degeneration and foci of apoptosis/necrosis were evident in the EtOH-fed mice (arrows). These morphological changes were markedly improved in the EtOH-fed+DNLA mice. Oil-red O staining further revealed extensive lipid droplets (red) in the EtOH-fed group (Figure 3B). Compared with the EtOH-fed group, the red areas in the EtOH-fed+DNLA group were much less, and the “red” intensity was roughly the same as that in the pair-fed group. 

### 3.3. DNLA Alleviated Chronic Alcohol-Induced Aberrant Gene Expression

#### 3.3.1. Gene Expression Pattern Analysis

**Figure 4:**
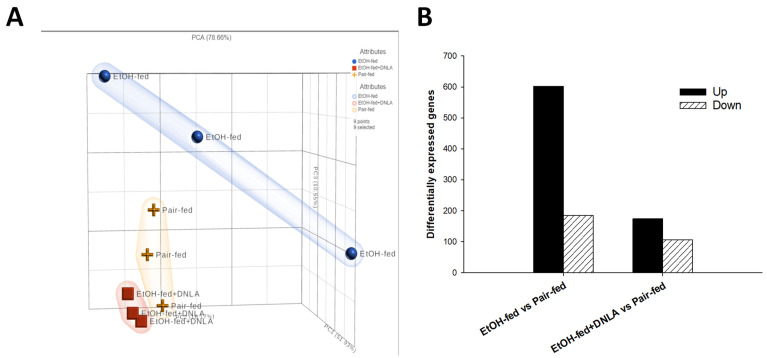
Differentially expressed gene (DEG) analysis. (**A**). Principal component analysis (PCA) of 9 samples (3/group). (**B**). The histogram of DEGs of EtOH-fed vs. pair-fed, and EtOH-fed+DNLA vs. pair-fed (Padj < 0.01). Upregulated genes are black bars, and downregulated genes are shaded bars.

Liver total RNA was subjected to RNA-Seq (Chongqing Weilang Biotechnology). FPKM (fragments per kilobase of exon per million fragments mapped) obtained from RNA-Seq (~22,500/sample) were transformed to gene raw counts (three samples/group). All gene counts were subjected to principal component analysis (PCA) (Figure 4A). The PCA value was 78.65 %. PC1 = 51.93%, PC2 = 16.17%, and PC3 = 10.55%. The distribution of the EtOH-fed group (orange cross) was obviously different from those of the other groups; distributions of the EtOH-fed+DNLA (red square) were apparently separated from the EtOH-fed group (orange cross); the pair-fed group (blue circle) and the DNLA (red square) were largely the same in pattern. Differentially expressed genes (DEGs) compared with the pair-fed group were subjected to the DESeq2 method with Padj < 0.01. The results showed that alcohol upregulated 602 genes and downregulated 185 genes; DNLA induced the expression of 174 genes and decreased the expression of 106 genes (Figure 4B). 

#### 3.3.2. Differentially Expressed Gene Analysis

**Figure 5:**
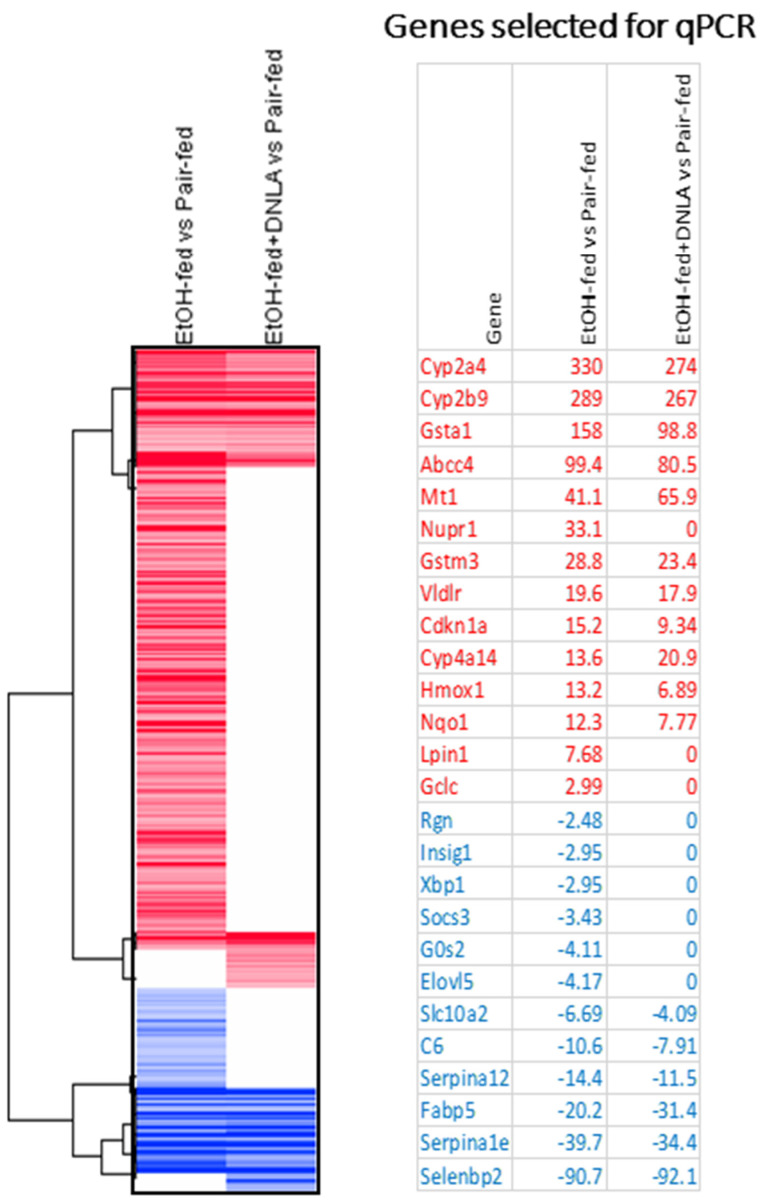
Heatmap of 2D clustering of DEGs among groups. Red represents increased gene expression and blue represents decreased gene expression. The scale bar represents +3/−3 Log2 fold change. Examples of up- and downregulated genes in representative clusters are shown on the right side.

Based on the above DEGs, two-dimensional clustering analysis was performed for comparison (Figure 5). The genes selected for qPCR verification are listed on the right side of the 2D clustering heatmap (Figure 5, sorted by fold changes). The upregulated genes are shown in red and the downregulated genes in blue. In general, qPCR verified these up- and downregulated genes and the effects. The complete list of genes is provided in Supplementary Table S2. 

#### 3.3.3. Ingenuity Pathways Analysis (IPA) of DEGs

**Figure 6:**
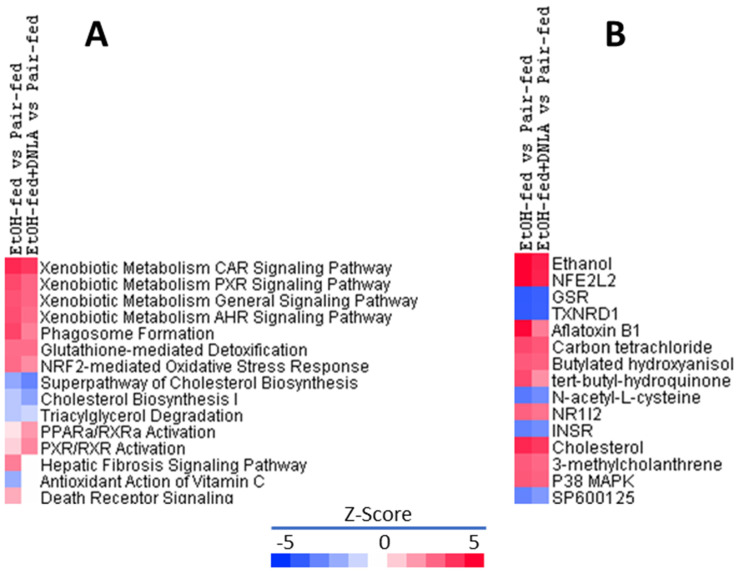
Ingenuity pathways analysis (IPA) of canonical pathways. (**A**) Top 15 canonical pathways from Ingenuity Pathways Analysis based on Z-score. (**B**) Identification of top 15 upstream regulators. Red indicates the upregulation and blue indicates the downregulation as compared to the control group.

The top 15 canonical pathways of IPA (Figure 6A) revealed that chronic alcohol mainly affected xenobiotic general metabolism pathways, particularly CAR, PXR, and AhR signaling pathways. All the enhanced Z-scores were attenuated by DNLA. DNLA also attenuated alcohol-induced glutathione-mediated detoxification and Nrf2-mediated oxidative stress responses, but DNLA appeared to be less effective in rescuing cholesterol biosynthesis and triacylglycerol degeneration. The EtOH-fed+DNLA group displayed more activation of PPARα and PXR/RXRα than the EtOH-fed group, suppressed the upregulation of hepatic fibrosis signaling and death receptor signaling, and prevented the downregulation of the antioxidant action of vitamin C in the EtOH-fed group. 

The IPA upstream regulator analysis was used to identify the upstream regulators that may be responsible for gene expression changes observed in the study. The top 15 upstream regulators are shown in Figure 6B. Ethanol was the most upregulated upstream regulator with a Z-score of 5.182 in EtOH-fed vs. pair-fed, and DNLA reduced it to 4.395. Nrf2 was ranked 2nd, followed by Nrf2-activating chemical aflatoxin B1, carbon tetrachloride, tert-butyl-hydroquinone, N-acetyl-L-cysteine, and Nrf2-targeted molecules GSR and TXNRD1. Compared to the EtOH-fed group, DNLA attenuated the increases in Z-scores, and ameliorated the downregulated Z-scores. NR1l2 (PXR) and PXR activator 3-methylcholanthrene were increased by the EtOH feeding but were attenuated by DNLA. Cholesterol and insulin receptor (INSR) were decreased by EtOH feeding, and DNLA had minor effects. Alcohol decreased JNK inhibitor SP6001125 and increased p38 MAPK, suggesting activation of MAPK pathways, and both were ameliorated by DNLA. 

#### 3.3.4. The Upregulated DEGs Verified by qPCR

**Figure 7:**
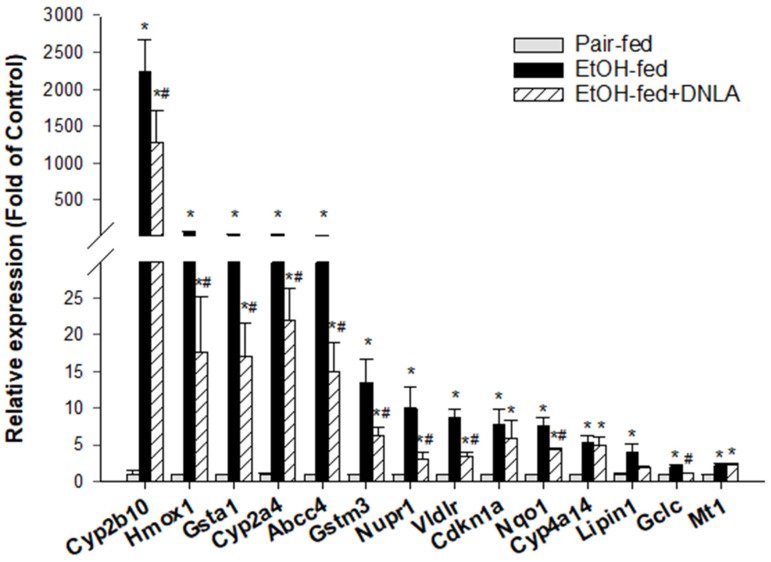
qPCR analysis of upregulated genes. Data are displayed as fold increase compared to pair-fed mice; mean ± SEM (n = 9). * Significantly different from pair-fed, *p* < 0.05. # Significantly different from EtOH-fed, *p* < 0.05.

Based on the IPA pathway analysis (Figure 6), typical biomarkers of CAR and PXR activation (*Cyp2b10*, *Cyp2a4*, and *Abcc4*) were firstly analyzed via real-time qPCR (Figure 7). The expression of *Cyp2b10* was increased 2245-fold by alcohol but attenuated to 1290-fold in the presence of DNLA. The expression of *Cyp2a4* was increased 35-fold by alcohol but attenuated by DNLA to 22-fold of the pair-fed. The expression of *Abcc4* was increased 32-fold by alcohol but decreased to 15-fold with DNLA. 

The commonly used oxidative stress biomarkers (*Hmox1*, *Nqo1*, and *Nupr*) and indicators of glutathione detoxification enzyme genes (*Gclc*, *Gsta1* and *Gstm3*) were further analyzed via the real-time qPCR (Figure 7). Chronic alcohol significantly stimulated the expression level of *Hmox1* expression to 71.5-fold but decreased it to 17.7-fold by DNLA. This trend also appeared in the expression of *Nqo1* and *Nupr1*. Both *Nqo1* and *Nupr1* were activated by alcohol (*Nqo1*, 7.6-fold, and *Nupr1*, 10-fold), but were ameliorated by DNLA (*Nqo1*, 4.4-fold, and *Nupr1*, 3.3-fold). The analysis of the expression of glutathione detoxification enzyme genes showed that the level of *Gsta1* mRNA expression was increased 42.5-fold by alcohol, and reduced to 17.2-fold with DNLA. Chronic alcohol induced *Gstm3* mRNA expression to 13.5-fold, but this was attenuated with DNLA to 6.3-fold. *Gclc* was increased 2.1-fold by alcohol but unchanged after DNLA. The alcohol-induced Nrf2-targted gene *Vldlr* (8.7-fold) was suppressed to 3.5-fold by DNLA. The expression of *Cyp4a14* was increased 5.5-fold and 5.0-fold in the EtOH-fed and EtOH-fed+DNLA groups, respectively. The expression of *Lipin1* was increased 4-fold by alcohol but alleviated to 2-fold after DNLA. The expression of *Cdkn1a* was increased by 7.8-fold by alcohol and attenuated by 6.0-fold by DNLA. The expression of *Mt1* was similarly increased by 2.3-fold in both EtOH-fed and EtOH-fed+DNLA groups. 

#### 3.3.5. The Downregulated DEGs Verified by qPCR

**Figure 8:**
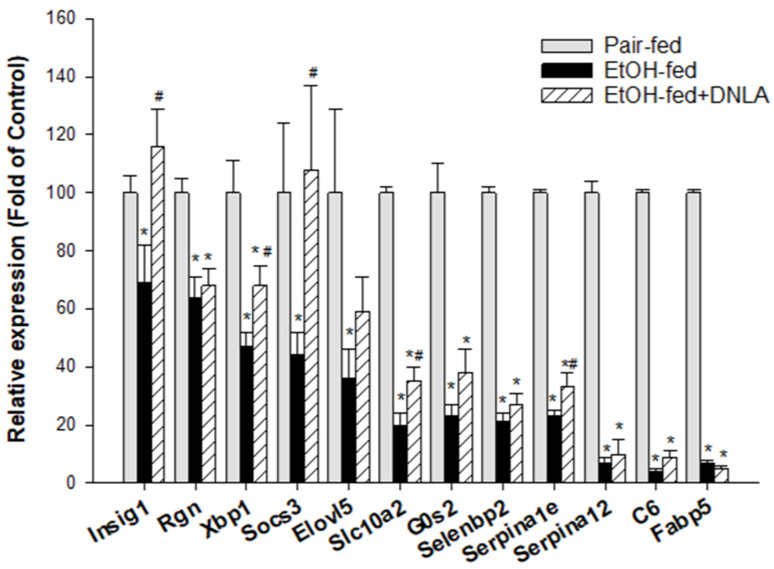
qPCR analysis of downregulated genes. Data are displayed as percentage of pair-fed, mean ± SEM (n = 9). * Significantly different from pair-fed, *p* < 0.05. # Significantly different from EtOH-fed, *p* < 0.05.

The downregulated genes were further analyzed via the real-time qPCR and are shown in Figure 8. The expression of *Insig1* was decreased to 70% and rescued by DNLA. The expression of *Rgn* was decreased to 65% regardless of DNLA treatment. The expression of *Xbp1* was decreased to 50% and rescued by DNLA to 70%. The expression of *Socs3* was decreased to 45%, and this decrease was prevented by DNLA. The expression of *Elovl5* was decreased to 36% and 60% of the original value by alcohol and DNLA, respectively. Angptl8 was decreased to 34% of the original value by alcohol and 36% in the EtOH-fed+DNLA group (data not shown). The expression of *Slc10a2* was decreased to 20% of the original value by alcohol but slightly increased to 35% by DNLA. The expression of *G0s2* was decreased to 23% and 38% of the original value by alcohol and DNLA. As for *Selenbp2*, the alcohol suppressed its level to 21% of the pair-fed level, and to 27% in EtOH-fed+DNLA. The expression of *Serpina1e* was decreased to 23% of the original value by alcohol and slightly recovered to 33% by DNLA. The expression of *Serpina12* was decreased to 7% by alcohol and 10% by DNLA, respectively. Alcohol severely decreased the level of *C6* to 4% of the original value, and it was 9% in the EtOH-fed+DNLA group; and the expression of *Fabp5* and Mup7 (data not shown) became 5–7% of the original levels in both groups. 

### 3.4. Western-Blot Analysis of Selected Proteins

**Figure 9:**
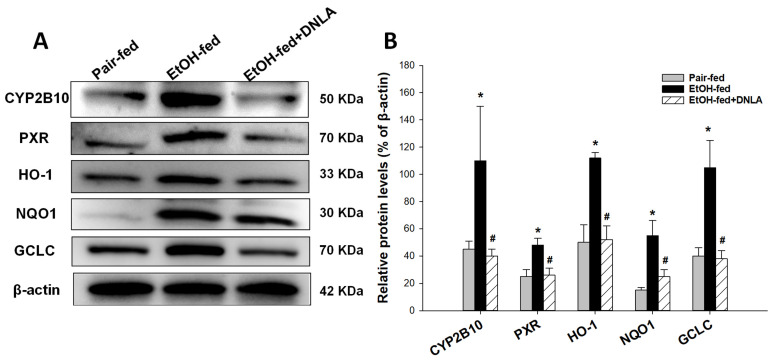
Western-blot analysis of selected proteins. (**A**) Representative Western-blot images. (**B**) Quantification of Western-blot intensity relative to β-actin. Data are mean ± SEM (n = 3). * Significantly different from pair-fed, *p* < 0.05. # Significantly different from EtOH-fed, *p* < 0.05.

To further verify gene expression results, the protein expression levels of CYP2B10, PXR, HO-1, NQO1, and GCLC proteins were examined by Western blot (Figure 9). The representative images are shown in Figure 9A, and the quantitations (n = 3) relative to β-actin are shown in Figure 9B. The results demonstrate that DNLA could reduce the over-expression of CYP2B10 (CAR), PXR, HO-1 (oxidative stress), NQO1, and GCLC (Nrf2) proteins induced by alcohol, which is consistent with gene expression findings.

## 4. Discussion

The protective effects of DNLA against chronic alcohol-induced fatty liver disease were evidenced by decreased serum triglycerides, and improved histopathology with less abundance of hepatic lipid droplets. Importantly, DNLA alleviated alcohol-induced differentially expression of genes. IPA showed that DNLA attenuated alcohol-induced oxidative stress and ameliorated xenobiotic metabolism through CAR, PXR, and Nrf2 signaling pathways. qPCR verified the selected up- and downregulated genes. To our knowledge, this study is among the first to demonstrate transcriptome changes in chronic alcohol liver injury and the beneficial effects of DNLA.

### 4.1. DNLA Protected against Chronic Alcohol-Induced Fatty Liver

The Lieber–DeCarli alcohol diet-induced alcoholic liver injury involves mild liver damage characterized by steatosis and mild inflammation [6,21], which is different from acute CCl_4_-induced liver injury with marked elevation of serum enzymes, hepatocyte necrosis, lipid peroxidation, mitochondrial dysfunction, and oxidative damage [15,16]. In the present study, increases in serum enzyme activities were mild with foci of apoptosis/necrosis, rendering a serum triglyceride increase and lipid accumulation in the liver the major pathologies. DNLA is not only effective at protecting against acute CCl_4_ hepatotoxicity [15,16], but also at protecting against chronic alcoholic liver injury. 

### 4.2. DNLA Alleviated Chronic Alcohol-Induced Aberrant Gene Expression

Principal component analysis (PCA) of RNA-Seq data is exploratory and for visualizing gene expression patterns [24]. The gene expression of the EtOH-fed group was apparently different from that of the pair-fed group, and DNLA brought the aberrant gene expression levels towards normal (Figure 4). Compared to pair-fed mice, chronic alcohol consumption produced 787 DEGs, and treatment with DNLA left only 280 DEGs. The 2-dimensional heatmap showed different clusters. A few gene clusters are provided as examples (Figure 5), and the entire list is provided in Supplementary Table S2. 

### 4.3. DNLA Attenuation of Overexpressed Genes

Canonical pathway analysis (Figure 6) revealed that alcohol mainly affects xenobiotic metabolism, particularly CAR, PXR, and Nrf2 signaling pathways, and the upstream regulators pointed to Nrf2, PXR, and oxidative stress as major targets. To verify IPA predictions, the CAR and PXR biomarkers (*Cyp2a4*, *Cyp2b10*, and *Abcc4*) were examined via qPCR (Figure 7). Alcohol dramatically increased these genes’ expression levels, which were attenuated by DNLA. CAR participates in glucose and lipid metabolism and plays important roles in fatty liver disease and diabetes. CAR-null mice showed increased sensitivity to chronic alcohol-induced liver injury; however, over-activation with CAR agonist TCPOBOP enhanced hepatotoxicity in both acute and chronic alcohol exposures [25]. PXR-null mice are resistant to alcohol-induced fatty liver and aberrant gene expression [26]. *Cyp2a4*, *Cyp2b10*, and *Abcc4* are all CAR and PXR target genes in response to alcohol [27,28]. Polyphenols (resveratrol and ellagic acid) protect against chronic alcohol-induced fatty liver in wild-type but not CAR-null mice [29]. Similarly, *Cyp2a5*-null mice exhibited enhanced alcoholic liver injury and hyperglycemia [4]. Thus, appropriate activation of CAR and PXR is beneficial as an adaptive response to alcohol exposure within the threshold range, but their overaction could aggravate the liver injury [6,25]. 

Hepatocyte-specific Nrf2 activation plays major roles in controlling steatohepatitis, fibrogenesis and carcinogenesis [30]. For example, *Penthorum chinense* Pursh protected against Lieber–DeCarli alcohol-diet-induced liver injury by upregulating Nrf2 and its downstream antioxidant protein *Ho-1* [18]. Curcumin attenuates chronic alcohol liver injury via induction of the Nrf2 pathway genes *Nqo1*, *Ho-1*, *SOD*, and *Gpx* through ERK/p38/Nrf2 antioxidant signaling pathways [19]; and green tea extract protected against alcohol-induced liver injury through the Nrf2 signaling pathway [20]. Nrf2 activation also induces the downstream *Vldlr* to protect against alcohol-induced oxidative stress and hepatocyte injury [31]. Ursolic acid, a triterpenoid and a strong Nrf2 activator, protected against chronic alcohol liver injury by inducing *Nqo1*, *Gclc*, and glutathione S-transferases [32]. Glutathione detoxification enzyme genes, including *Gsta1*, *Gstm3*, *Gstm1*, *Gstp1*, *Gclm*, and *Gclc*, play important roles in protecting against alcoholic liver damage and diseases [33,34]. Activation of Nrf2, glutathione detoxification systems could be considered as an adaptive response to alcohol exposure, and their over-activation implies severe liver injury. The attenuation of the above genes by DNLA indicates reduced oxidative damage to the liver. The protein analysis of CYP2B10 (CAR), PXR, HO-1 (oxidative stress), NQO1, and GCLC (Nrf2) further supported the gene expression findings. Additionally, the PARP pathways are implicated in alcohol liver injury [35], and one PARP-pathway-targeting gene, glycerol kinase 5 (*CK5*), was increased 8.99-fold by alcohol but was moderated by DNLA (Supplementary Table S2). 

### 4.4. DNLA Ameliorated Downregulated Genes 

Alcohol-downregulated genes from RNA-Seq and IPA analyses were verified by qPCR. Insulin-induced gene 1 (Insig1) plays roles in maintaining lipid homeostasis, but *Insig1*^−/−^ mice could promote liver lipid remodeling [36]. The expression of regucalcin (*Rgn*) is stimulated by insulin in the liver, and decreased *Rgn* is associated with fatty liver disease in humans [37]. The liver-specific G(0)/G(1) switch gene (*G0s2*) could promote insulin resistance and exacerbate steatosis and pair-fed adipose-liver fatty-acid influx [38]. The decreases in these genes were rescued by DNLA to various extents. Suppressors of cytokine signaling (*Socs*) were downregulated in response to metabolic disruption [39]. X-box binding protein 1 (*Xbp1*) regulates liver lipogenesis [40] by inducing fatty acid synthesis genes such as fatty acid elongase-5 (Elovl5) [41]. Serpin family genes (*Serpina12*, also called vaspin and *Serpina1e*) are protective against alcohol-induced steatosis but are decreased in in alcoholic patients and animals [42]. Downregulation of *Serpina1e* is also associated with a high-fat diet proteomic phenotype [43]. The decreases in these genes were partially ameliorated by DNLA. Mouse major urinary proteins (Mups) are a family of proteins that are expressed in the liver and excreted in urine. Mups are severely downregulated by dietary restriction [44]. More than 10 Mups and fatty acid-binding proteins, such as *Fabp5*, were severely downregulated by alcohol in this study but were unaffected by DNLA. In general, the qPCR confirmed RNA-Seq results. The liver is a crucial organ for lipogenesis, gluconeogenesis, and cholesterol metabolism, which are regulated by nuclear receptors and other factors [45]; and the downregulation of these metabolism genes could be an adaptive response. However, most of downregulations were too dramatic to be rescued by DNLA. 

### 4.5. Beneficial Effects of DNLA 

DNLA has anti-aging, anti-diabetes, and anti-hyperlipidemia activities [10] through the regulation of hepatic metabolism genes to maintain the lipid homeostasis [13,14]. For toxicity insults, such as exposure to CCl_4_ or chronic alcohol, however, reducing oxidative damage is perhaps the predominant mechanism to keep hepatocyte alive [15,16]. Multiple mechanisms could be involved in DNLA protection, including the activation of Nrf2/ARE pathways [15], the prevention of mitochondrial damage [16], and modulation of mitochondrial oxidative phosphorylation to produce adaptive responses [17]. The current study revealed that the attenuation of the over-activation of CAR, FXR, and Nrf2 signaling pathways is important in the protection against alcoholic fatty liver disease. 

## 5. Conclusions

In summary, the present study clearly demonstrated that DNLA is able to confer protective effects against chronic alcoholic fatty liver and reverse the expression of genes involved in some key signaling pathways at the pair-fed level. In particular, over-expressed *Cyp2a4*, *Cyp2b10*, and *Abcc4* were dramatically reduced, and over-expression of oxidative-stress-related genes *Hmox1*, *Gsta1*, *Gstm3*, *Nupr1*, *Lipin1*, *Cdkn1a*, *Nqo1*, *Vldlr*, and *Gclc* was greatly attenuated. DNLA also rescued alcohol-suppressed *Insig1*, *Xbp1*, *Socs3*, *Slc10a2*, and *Serpina1e*, but had little effect on the downregulation of some metabolism-related genes, suggesting the protection mechanisms of DNLA may be mainly attributed to decreased oxidative stress, through modulating nuclear receptor CAR-, PXR-, and Nrf2-mediated gene expression pathways. 

## Data Availability

The data underlying this article are available in the article and in its online supplementary material (Supplementary Table S1 (Primer sequence for qPCR) and Table S2 (2D-dimentional analysis of differentially expressed gene list)).

## Supplementary Materials

The following supporting information can be downloaded at: https://www.mdpi.com/article/10.3390/biomedicines10112800/s1, Supplementary Table S1: Primer sequences; Supplementary Table S2: 2D-cluster of DEGs.
